# Beta-Boswellic Acid and Ethanolic Extract of Olibanum Regulating the Expression Levels of *CREB-1* and *CREB-2* Genes

**DOI:** 10.22037/ijpr.2019.1100665

**Published:** 2019

**Authors:** Asiyeh Jebelli, Mohammad Khalaj-Kondori, Mortaza Bonyadi, Mohammad Ali Hosseinpour Feizi, Mohammad Rahmati-Yamchi

**Affiliations:** a *Department of Animal Biology, Faculty of Natural Science, University of Tabriz, Tabriz, Iran.*; b *Department of Clinical Biochemistry, Faculty of Medicine, Tabriz University of Medical Sciences, Tabriz, Iran. *; c *Drug Applied Research Center, Tabriz University of Medical Sciences, Tabriz, Iran.*

**Keywords:** Memory, Olibanum, Beta-boswellic acid, CREB-1, CREB-2

## Abstract

Physiological studies confirm improvement of memory by Olibanum, a resin from *Boswellia* species, while little is known about the molecular mechanism by which it affects memory performance. Two master transcription factors, CREB-1 and CREB-2, regulate downstream memory-related genes expression, leading to the long-term memory potentiation. This study addresses the effects of Beta-boswellic acid (β-BA), the main ingredient of Olibanum, and ethanolic extract of the resin from *Boswellia serrata* on the expression of *CREB-1* and *CREB-2* genes in B65 cell line. B65 cells were treated with β-BA or ethanolic extract of Olibanum in different doses and time intervals and the cell viability/toxicity was measured by MTT assay. Total RNA was extracted from the treated and untreated control cells and cDNA was synthesized. The expression levels of *CREB-1 *and *CREB-2 *genes were quantified by Real-time PCR. MTT assays revealed 50% inhibitory concentrations of 42.05, 29.63, and 21.78 μg/mL for ethanolic extract of Olibanum and 89.54, 44.05, and 21.12 µM for β-BA at 24, 48, and 72 h time intervals respectively. Both β-BA and ethanolic extract of Olibanum altered *CREB-1 *and *CREB-2 *gene expression levels in time-dependent but not in dose-dependent way. However, β-BA showed stronger and more stable effects. The expression levels of the both genes followed an alternate upregulation and downregulation pattern, but in opposite directions, in response to the both solutions with the progress of time. These results suggest that Olibanum possibly improves memory performance, at least partially, by regulating the levels of CREB-1 and CREB-2 transcription factors via positive/negative-feedback loops.

## Introduction

Olibanum or frankincense is a herbal product which is produced by different species of the family *Burseraceae* such as *Boswellia serrate *(1). Its therapeutic effects on different diseases such as chronic inflammatory diseases (2-4), cancers (5-8), and diabetes (9, 10) have been documented. Moreover, there are lines of physiological studies highlighting its positive effects on the learning and memory procedures. For example, its administration during pregnancy and lactation increased hippocampal neurons number and improved off springs’ short- and long-term memory performance (11, 12). Further, it prevented learning and memory deficit induced by hypothyroidism in adult rats (13). Some protective and/or therapeutic effects of Olibanum on Alzheimer’s disease (AD) and epilepsy were reported in rat models of AD (14-16) or epilepsy (17). All these and other (18, 19) studies imply a memory-boosting effect for Olibanum which has been investigated mainly from physiological point of view while its molecular aspect remained extensively elusive. 

Even elementary forms of learning have distinct short-term and long-term stages of memory storage (20, 21). While short-term memory is not dependent on protein synthesis, plenty of evidence from different model systems indicates that the formation of long-term memory depends on it (22, 23). The CREB/ATF superfamily includes some structurally-related transcription factors which function at diverse procedures including cell proliferation, survival, and apoptosis (24). The cAMP responsive element binding protein (CREB), a member of CREB/ATF superfamily, binds to the cAMP response element (CRE) sites in the promoter region of different genes and thereby regulate the transcription of downstream genes (25, 26). There are two CREB isoforms, CREB-1 and CREB-2, which both express in different tissues especially in the hippocampus. The CREB-1 acts as a memory-boosting factor where it is a crucial component of long-term memory formation and its function leads to the growth of new synapses (27, 28). On the other hand, CREB-2 is a memory-suppressing factor. It represses CREB-1 mediated transcription, by binding to the* CREB-1* promoter and inhibition of its transcription as well as by inactivation of CREB-1 proteins, via binding to and formation of heterodimer with CREB-1, or by activating the transcription of memory-suppressor genes (25, 28-30). 

Olibanum resin contains terpene residues, including monoterpenes (Limonene), Diterpenes (cembrene A, Incensole, incensole-acetate, serratol, and iso-serratol) and Triterpenes (Boswellic, lupeolic, roburic and tirucallic acids) among which the boswellic acids (BAs) are highly specific biomarker for *B.serrata*. Therapeutic effects of Olibanum are attributed to BAs of which the Beta-boswellic acid (β-BA) is the important one (31). To pave a sight on the molecular mechanism by which Olibanum or its ingredients promote the learning and memory, we analyzed the effect of β-BA and ethanolic extract of Olibanum on the expression of the two master memory-related genes, *CREB-1* and *CREB-2*, in the B65 cell line. this CNS-derived cell is a laboratory cell model for studying the molecular events in neuronal cells (32). B65 cells have the same features as serotonergic neurons and have been already used for studying of CREB-related pathway under different conditions (33-35).

## Experimental

*Reagents and Materials*

Fetal bovine serum (FBS), **c**ell culture media RPMI 1640 and Pen-strep antibiotic were purchased from Gibco® Life Technologies (Waltham, MA, USA). Olibanum resin used in this study was purchased from Mother Herbs (Delhi, India). Isopropanol, ethanol, and chloroform were obtained from Merck (Kenilworth, NJ, USA). Reverse transcriptase, random hexamer, deoxy nucleotide triphosphates (dNTPs), and RNase inhibitor were provided from Thermo Fisher** S**cientific (Waltham, MA, USA). SYBR® Green PCR Master Mix was available from Amplicon (Brighton, UK). β-BA (80342-5MG) and all other chemicals were obtained from Sigma-Aldrich (St Louis, MO, USA).

*Cell line*

Rat neuroblastoma B65 cell line was obtained from the National Cell Bank of Iran (Pasteur Institute of Iran, Tehran, Iran). Cells were grown in **c**ell culture media RPMI 1640 which was supplemented with 10% FBS inactivated at 56 °C and 1% Pen-strep antibiotic (0.25% trypsin-EDTA) and cultured at 37 °C in a humidified incubator containing 5% CO2. The B65 cells were detached using 0.1% trypsin in Ca^2+^/Mg^2+^-free PBS/0.025% EDTA.

*Preparation of the ethanolic extract of Olibanum*

For preparation of the ethanolic extract of Olibanum, a total of 50 grams of the dry powder of Olibanum has been solved in 200 mL of ethanol. After two days, the solution was centrifuged and the supernatant was evaporated in a Soxhlet extractor to obtain a sticky component. Finally, the extract was solved in organic solvent dimethyl sulfoxide (DMSO) and stored at 4 °C.

*Cell viability/toxicity assay*

The B65 cells were plated into 96-well microplates in a density of 7×10^3^ cell/well. After 24 h incubation at 37 °C, cells were treated with concentrations 10, 25, 40, 55, 70, and 85 µg/mL or 0.5, 1, 25, 50, and 100 µM of ethanolic extract of Olibanum or β-BA respectively for 24, 48 and 72 h at 37 °C with 5% CO2. All treatments were made at triplicate format. Then, 20 μL MTT (3-(4, 5-dimethylthiazol-2-yl)-2, 5-diphenyltetrazolium bromide) solution (5 mg/mL) was added to each well, and the cells were incubated at 37 °C for 4 h. The medium was then removed from the wells, and 150 μL DMSO was added to each well, shaking at room temperature for 15 min to dissolve the produced intracellular formazan complexes. Next, the absorbance was read by ELISA reader at 570 nm and the viability percentage was calculated as viability of treated cells relative to the untreated control. Finally, the 50% inhibitory concentrations (IC_50_) of ethanolic extract of Olibanum and β-BA for corresponding time intervals were calculated by Graph Pad Prism software from the curve generated using a variable slope non-linear regression curve fit.

*Cell treatments with ethanolic extract of Olibanum and β-BA for gene expression studies*

The B65 cells were seeded into 6-well plates containing RPMI 1640 medium supplemented with 10% FBS in a density of 2×10^5^ cell/well and incubated for 24 h. The cells were starved for 6 h in the medium with 4% FBS before treatments. Then the cells were treated with ethanolic extract of Olibanum at concentrations of 2 or 20 µg/mL and β-BA at concentrations of 1 or 10 µM. The cells were incubated at 37 ºC with 5% CO2 for 12, 24, 48, and 72 h. All treatments were made at duplicate format and repeated for two times. 

*RNA extraction*

The wells were washed with phosphate-buffered saline (PBS) and the cells were removed from the plate with 0.1% trypsin and collected in 2 mL vials. Then, the cells were resuspended in 1 mL lysis buffer RNX-PLUS for 5 min, and mixed with 200 µL of chloroform. The vials were put on ice for 5 min and centrifuged at 12000 rpm for 15 min. The aqueous phase was transferred into new 1.5 mL vial and an equal volume of isopropanol was added to precipitate RNA. The total RNA was precipitated at 12000 rpm for 15 min, washed with 75% ethanol, and the RNA pellet was dissolved in RNase-free water. The integrity of extracted RNA was evaluated by agarose gel-electrophoresis and quantified by NanoDrop 1000 Spectrophotometer (Thermo Scientific, USA).

*cDNA synthesis and quantitative Real-time PCR*

About 1500 ng total RNA was treated by 1 U DNase I enzyme at 37 °C for 30 min. Then, EDTA was added and incubated at 65 °C for 10 min to inactivate DNase I. Next, first-strand cDNA synthesis was performed using M-MuLV reverse transcriptase. In a 0.5 mL tube, DNase-I treated RNA was mixed with 4 μL 5X RT master mix (composed of 250 mM Tris-HCl, 375 mM KCl, 15 mM MgCl _2_), 1 mM dNTPs, 8 μM random hexamer, 1.5 U reverse transcriptase and RNase inhibitor in a total volume of 20 μL and incubated at 42 °C for 60 min.

Real-time PCR was performed with the Rotor-Gene^TM^ 6000 (Corbett Life Science, Australia). The PCR reactions were carried out in a total volume of 10 µL using 5 µL SYBR® Green PCR Master Mix (2X), 0.3 µL forward and reverse primers mix (10 pmol), 3 μL diluted cDNA and 1.7 µL ddH_2_O. The primers used were: *GAPDH* forward: 5՜-CATAGACAAGATGGTGAAGGTCG-3՜ and reverse: 5՜- CCGTGGGTAGAGTCATACTGG-3՜ as internal control, *CREB-1* forward: 5՜- AAGACTTTTCTCCGGAACTCA-3՜ and reverse: 5՜-TCTTCTTCAATCCTTGGCAC-3՜ and the *CREB-2* forward: 5՜-TATGGATGGGTTGGTCAGT-3՜ and reverse: 5՜- GTTTCCAGGTCATCCATTC-3՜. The reactions were followed as 95 °C for 15 min and 40 cycles of 95 °C for 15 Sec, 60 °C for 15 Sec and 72 °C for 15 Sec. The Data were analyzed with Rotor-Gene 6000 series software 1.7 and the relative expression levels of genes were calculated using 2^-ΔCT^ method.

*Statistical analysis*

The analysis of MTT data was performed using Graph Pad Prism version 6 and expressed as the mean ± SD from three independent experiments. Real-time PCR data were analyzed using SPSS version 19 (SPSS Inc., Chicago, MI, USA) and the independent Student’s t-test program. A p-value of 0.05 was set as the level of significance. 

## Results

*Viability/toxicity assay*

**Figure 1:**
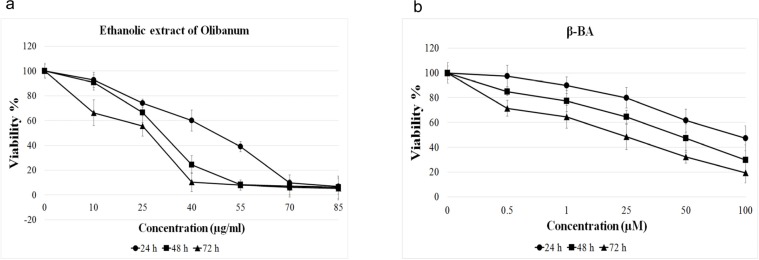
MTT assays. B65 cells cultured in plate were treated with different doses of ethanolic extract of Olibanum (a) or Beta- boswellic acid (b) for 24, 48 and 72 h in triplicate format. Then, MTT solution was added to plate and the cells were incubated for 4 h. The absorbances were read by ELISA reader at 570 nm. Both ethanolic extract of Olibanum and β-BA decreased the cell viability in a dose- and time- dependent manner. The Data are means ± SD of three independent repeats

To elucidate toxic effects of ethanolic extract of Olibanum and β-BA on B65 cells, we measured the viability parameter of the treated cells using MTT assay. Figure 1 illustrates the MTT assay results. It shows that both ethanolic extract of Olibanum and β-BA have dose- and time- dependent effects on viability of B65 cells. We obtained IC_50_ of 42.05, 29.63, and 21.78 μg/mL for ethanolic extract of Olibanum and 89.54, 44.05, and 21.12 µM for β-BA at 24, 48, and 72 h time intervals respectively. Based on these results, to avoid toxic effects of treatments at gene expression analysis experiments, we used two lower doses of ethanolic extract of Olibanum (2 and 20 μg/mL) and β-BA (1 and 10 μM) for all the following gene expression analysis tests.

*Effects of ethanolic extract of Olibanum on the expression of CREB-1 and CREB-2 genes*

**Figure 2:**
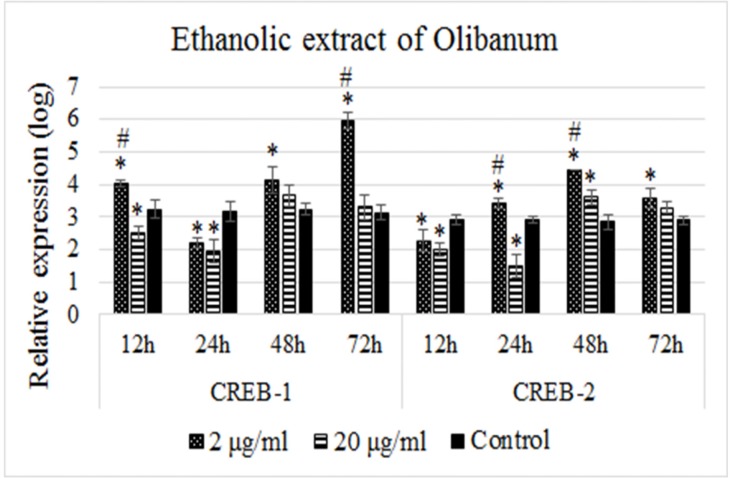
Relative expression profiles of the *CREB-1 *and *CREB-2 *genes in response to treatments with the ethanolic extract of Olibanum. B65 cells were seeded in plates and treated with 2 and 20 µg/mL doses of the ethanolic extract of Olibanum for four time intervals in duplicate format. The expression levels of *CREB-1 *and *CREB-2 *were measured with Real-time PCR and normalized using GAPDH as internal control. The relative expressions were calculated using 2-∆ct method and analized with independent Student’s t-test. The results of expression studies represented that 2 µg/mL dose of ethanolic extract of Olibanum regulated the expression of *CREB-1 *and *CREB-2 *in an opposite manner. However, 20 µg/mL dose of extract showed dose-independent effects on the expression of both genes. A p-value of 0.05 was set as the level of significance. The data are mean±SD of two independent repeats. * p0.05 < , vs. control group. # p0.05 < difference between treatment groups

B65 cells were treated with concentrations 2 and 20 µg/mL of the ethanolic extract of Olibanum for 12, 24, 48, and 72 h and the expression of *CREB-1* and *CREB-2* genes were measured by quantitative Real-time PCR. Non-treated cells were used as controls. Figure 2 illustrates the relative expression profiles of the *CREB-1* and *CREB-2* in different time intervals. As Figure 2 shows, treatment with 2 µg/mL of the ethanolic extract of Olibanum resulted in significant upregulation of *CREB-1* at the time point of 12 h, downregulation at 24 h followed by time-dependent upregulations at 48 and 72 h. But, the effect of this dose on the *CREB-2* expression was opposite to *CREB-1* at corresponding time intervals. *CREB-2* expression was significantly downregulated at 12 h, upregulated at 24 h and 48 h, but decreased at 72 h time points. On the other hand, we did not observed this type of expression profile changes for *CREB-1* or *CREB-2* in the treatments with 20 µg/mL dose of the ethanolic extract of Olibanum. Instead, we observed decreased expression levels of both genes at 12 h and 24 h, but insignificant increased expression levels at 48 h and 72 h time intervals (Figure 2). In other words, the ethanolic extract of Olibanum showed dose-independent effects on the expression of *CREB-1* and *CREB-2* genes.

*Effects of β-BA on the expression of CREB-1 and CREB-2 genes*

**Figure 3:**
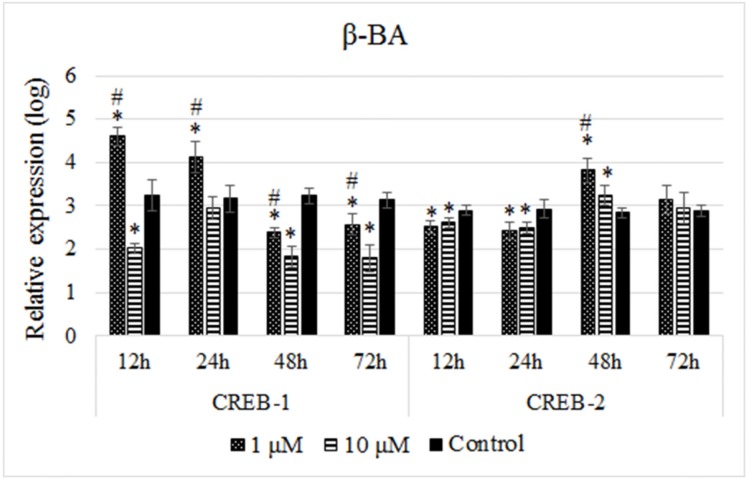
Relative expression profiles of the *CREB-1 *and *CREB-2 *genes in response to treatments with the Beta-boswellic acid. As performed for the ethanolic extract of Olibanum, B65 cells were treated with two doses of the β-BA in four time intervals and the expression of both genes were quantified. The relative expressions were calculated using 2-∆ct method and analized with independent Student’s t-test. The cell treatment with 1 µM dose of the β-BA resulted upregulation and then downregulation of *CREB-1. *However, the effect of this dose for *CREB-2 *expression was opposite to *CREB-1*. The results obtained from expression experiments with 10 µM dose of β-BA indicated that the effects β-BA on the expression of *CREB-1 *and *CREB-2 *genes were dose-independent. A p-value of 0.05 was set as the level of significance. The data are mean±SD of two independent repeats. * p0.05 < compared with control. #p0.05 < difference between two treatment groups

Like experiment performed for the ethanolic extract of Olibanum, B65 cells were treated with two doses of the β-BA (1 µM and 10 µM) for four time intervals and the expression of both genes were quantified. Treating the cells with the 1 µM dose of the β-BA resulted in upregulation of *CREB-1* at the time points of 12 h and 24 h, while a downregulation at 48 h and 72 h in comparison with the controls (Figure 3). Conversely, these treatments resulted in downregulation of *CREB-2* at 12 h and 24 h but a significant upregulation of it at 48 h which was followed by an insignificant expression change at 72 h in comparison with the untreated cells (Figure 3). On the other hand, 10 µM dose of β-BA significantly reduced the expression of *CREB-1* at all time points while it reduced the expression of *CREB-2* at 12 and 24 h which was followed by increasing levels at 48 h and 72 h time points (Figure 3). These results indicated that the effects of β-BA on the expression of *CREB-1* and *CREB-2* genes were dose-independent.

## Discussion

The effect of Olibanum on the learning and memory has been investigated by several studies which specified a memory promoting role for this herbal product (13, 14, 17-19, 36). However, the molecular mechanism by which it affects the memory is mostly elusive. To pave some light on its memory enhancement molecular mechanism as well as comparing the effects of ethanolic extract of Olibanum and beta-boswellic acid (β-BA), one of the main ingredients of it, we treated the B65 cells with different doses of the ethanolic extract of Olibanum or β-BA and quantified the expression levels of *CREB-1* and *CREB-2*. Our results revealed that the ethanolic extract of Olibanum as well as β-BA modulate the expression of *CREB-1* and *CREB-2* in a way resulting in memory improvement. 

**Figure 4.**
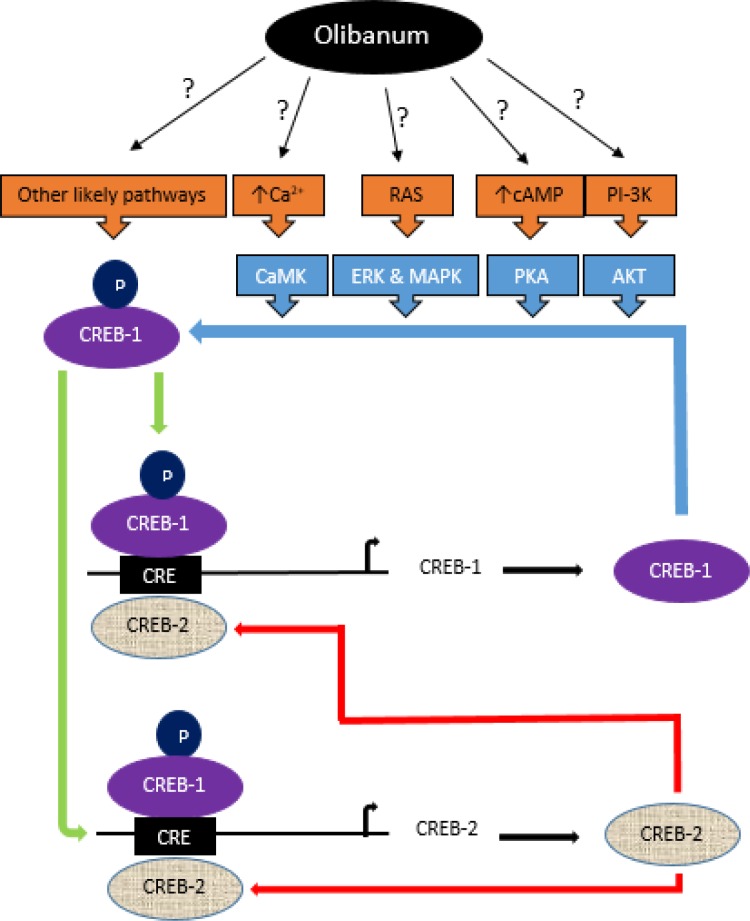
A putative model of positive and negative loops in the regulation of *CREB-1 *and *CREB-2 *expression and likely pathways by which Olibanum could control these loops. CREB-1, main memory transcription factor, regulates the expression of several genes such as its own and *CREB-2 *by binding to CRE sequences in the promoter of genes. To be activated, CREB-1 should be phosphorylated by kinases CaMK, ERK, PKA, AKT and MAPK. Each of these kinases is belong to a pathway which activated by different extracellular signals. Activated CREB-1 induces its own as well as *CREB-2 *transcription in a positive-feedback loop. Increased levels of CREB-2 promote the negative-feedback loop and repress the expression of its own and *CREB-1*. Olibanum is able to increase the expression of *CREB-1 *and then *CREB-2 *likely via phosphorylation and activation of CREB-1. However, the precise mechanism by which Olibanum induces signaling pathways is unclear and needs further investigation

CREB-1 and CREB-2 are two related transcription factors which their expression levels are regulated via two positive- and negative-feedback loops where CREB-2 is the repressor of CREB-1 (37-39). In the positive-feedback loop, CREB-1 induces its own as well as *CREB-2* transcription which leads to increased levels of CREB-1 and CREB-2 (39-43). Increased levels of CREB-2 promote the negative-feedback loop where CREB-2 binds to the CRE sequences at its own and also, the *CREB-1* promoters lead to both their repression (39). Interestingly, the results of our experiments follow this pattern. As the Figures 2 and 3 show, firstly, CREB-1 level increases in response to the treatments while the level of CREB-2 is low. But with the progress of the time the level of CREB-2 increases which leads to decreasing of the CREB-1 level. This condition oscillates until reaching to an equilibrated state. 

In addition, the results of the present study are in line with the results of the previous study by Moussaieff *et al*. where they showed the upregulation of the brain-derived neurotrophic factor (BDNF), which acts upstream of the CREB pathway (44), in response to the treatments with Insensole acetate, another important ingredient of the Olibanum (45). Our previous study also demonstrated upregulation of BDNF in response to the aqueous extract of Olibanum; however, we did not observe any significant changes in the *CREB* expression (46). Although the last observation seems contradictory with the results of the present study, but in fact, it is not conflicting in real, since the expression analysis was carried out at the end of the treatment period (after 28 days), when the expressions of *CREB-1* and *CREB-2* probably reach to an equilibrated state. This fact can be also seen in the present study, comparing just the expression levels of *CREB-1/CREB-2* with the controls at the time point of 72 h in Figure 3. 

Comparing the effects of ethanolic extract of Olibanum with the effects of β-BA revealed that both solutions affected the expression of *CREB-1* and *CREB-2* genes in the same manner, however; the effects of β-BA were more stable which need more time for alternating. For example, treatments with β-BA led to upregulation of *CREB-1* which lasted for two time points 12 h and 24 h, also its downregulation lasted for the two late time points 48 h and 72 h (compare the Figures 2 and 3); however, successive up and down levels of expressions were observable. The other noticeable fact was dose-independency of the observed effects for both solutions. Both 10 µM of the β-BA and 20 µg/mL of the ethanolic extract of Olibanum had almost stable repressing effects on *CREB-1* and *CREB-2* expressions. This observation might be critical, highlighting possible adverse effect of high doses of Olibanum on the memory performance, which needs further investigation.

These results strongly imply that the ethanolic extract of Olibanum and β-BA affects the memory performance at least partly via the CREB pathway. However, the precise mechanism by which Olibanum induces CREB pathway reminds unclear and needs to be clarified in future studies. Figure 4 indicates a putative model of Olibanum effect on the control of signaling pathways relating to memory. 

In conclusion, we established the modulatory effect of Olibanum on the expression of *CREB-1* and *CREB-2*, the two master genes in the memory formation and storage. Olibanum could regulate the expression of these genes in the nouroblastoma B65 cell line; however, this effect was not dose-dependent. Our findings are in line with previous studies confirming the positive effects of Olibanum in the memory performance and demonstrate that this botanical has likely the ability to target genes/pathways relating to memory. Moreover, these results highlight the potential of Olibanum as a therapeutic agent for cognitive disorders. 
